# Impact of sarcopenia on postoperative pulmonary complications after gastric cancer surgery: A retrospective cohort study

**DOI:** 10.3389/fsurg.2022.1013665

**Published:** 2023-01-06

**Authors:** Xiaofang Zhang, Chaoyi Deng, Qianyi Wan, Rui Zhao, Liping Han, Xiao Wang

**Affiliations:** ^1^ Department of Anesthesiology, West China Hospital, Sichuan University, Chengdu, China; ^2^ Laboratory of Anesthesia and Critical Care Medicine, National-Local Joint Engineering Research Center of Translational Medicine of Anesthesiology, West China Hospital, Sichuan University, Chengdu, China; ^3^ Department of Gastrointestinal Surgery, West China Hospital, Sichuan University, Chengdu, China

**Keywords:** sarcopenia, skeletal muscle index (SMI), anesthesia, postoperative pulmonary complications (PPCs), gastric cancer

## Abstract

**Background:**

Few studies have investigated the relationship between sarcopenia and postoperative pulmonary complications (PPCs) after gastric cancer surgery. This study aimed to explore the impact of sarcopenia on PPCs in patients who had undergone gastric cancer surgery.

**Methods:**

We included patients who underwent a transabdominal radical gastrectomy between June 2016 and October 2020. Patients were divided into two groups according to the median prevalence rate of lumbar triplane skeletal muscle index (L3 SMI): sarcopenia group (≤37.5% percentile in male and female group) and non-sarcopenia group (>37.5% percentile in male and female group). Baseline characteristics, intraoperative and postoperative conditions, pulmonary complications, and overall complications were compared between the two groups. The primary outcome was the incidence of PPCs. The secondary outcomes were overall postoperative complications and length of stay (LOS).

**Results:**

Among the 143 patients included, 50 had sarcopenia and 93 had not. Compared to the non-sarcopenia group, the sarcopenia group had a higher the incidence of PPCs (22.0% vs. 8.6%, *P* = 0.024). The incidence of overall postoperative complications in the sarcopenia group was higher than that in the non-sarcopenia group (36.00% vs. 20.43%, *P* = 0.043). There was no significant difference in the LOS between the two groups.

**Conclusions:**

Our research indicates that sarcopenia, preoperative comorbidities, and longer duration of intraoperative oxygen saturation <95% were risk factors for PPCs. Sarcopenia is an independent risk factor for postoperative complications. Given that our results provided a correlation rather than causation, future prospective randomized trials are needed to confirm the relationship between sarcopenia and prognosis.

## Introduction

Sarcopenia is a complex age-related syndrome characterized by progressive and generalized loss of skeletal muscle mass and function (1, 2), with the potential for physical disability, loss of independence, and adverse consequences, such as death (3, 4). The etiology of sarcopenia may be related to skeletal muscle disuse, endocrine changes, chronic wasting disease, systemic inflammatory responses, insulin resistance, and malnutrition (5, 6). Excessive inflammatory responses and chronic wasting disease largely contribute to sarcopenia, especially in patients with cancer (7). Thus, sarcopenia and cancer are causally related. Patients with gastric cancer usually have a certain degree of anorexia and underlying metabolic changes such as increased energy consumption, catabolism, and inflammation. These direct effects are exacerbated by the combined effects of chemotherapy and major gastrectomy, resulting in decreased nutrient intake. Decreased nutritional intake in patients with gastric cancer can further aggravate the occurrence and development of sarcopenia (8, 9).

Gastric cancer is the fifth most common cancer and third leading cause of cancer-related deaths worldwide (10). Studies have shown that sarcopenia is an independent factor for postoperative complications and overall survival in patients with gastric cancer (11). Studies have shown that sarcopenia is very common in older people, with a prevalence of 5%–13% in people aged 60–70 years and 11%–50% in those aged >80 years of age. Large differences in prevalence are related to differences in the measurements and cutoffs used to define sarcopenia (12). The prevalence of sarcopenia among community residents in China was 4.8% among women and 13.2% among men aged ≥70 years (13). The prevalence of sarcopenia in patients with cancer has significantly increased by approximately 35.7% (14). With further aggravation of population aging, the number of older patients with gastric cancer will gradually increase (15). Surgery remains the most important treatment for gastric cancer (16). However, the high incidence of postoperative complications and low survival rate in such patients have always been a concern for clinicians (17, 18). Postoperative complications have been shown to affect overall survival (19). Predicting the risk of postoperative complications and how to better intervene in order to reduce postoperative complications have become the focus of attention. Previous studies have shown that patients with sarcopenia have a higher risk of postoperative complications, longer length of stay (LOS), and higher hospital costs than patients without sarcopenia (20). Among the postoperative complications, anastomotic leakage and pulmonary complications have the greatest influence on postoperative mortality and prolonged LOS (21). Anastomotic leakage has decreased with improvements in surgical techniques. Pneumonia or lung-related complications are the most common postoperative complications in individuals under 80 years of age (22). Accurately predicting the risk of complications and actively preventing and doing everything possible to reduce the occurrence of postoperative pulmonary complications (PPCs) have become the focus of surgeons and anesthesiologists. However, few studies have investigated the relationship between sarcopenia and PPCs after gastric cancer surgery. In this study, we aimed to investigate the impact of sarcopenia on PPCs in patients undergoing gastric cancer surgery and to identify other risk factors for post-operative pneumonia.

## Materials and methods

### Study design and patients

This single-center, retrospective cohort study used data obtained from the discharge medical records of patients undergoing gastrointestinal surgery from June 2016 to October 2020 in the Department of Gastrointestinal Surgery, West China Hospital, Sichuan University, Chengdu, China. This study was approved by the Biomedical Research Ethics Committee of West China Hospital, Sichuan University, and was registered at www.chictr.org.cn (ChiCTR1900026578).

### Inclusion and exclusion criteria

The inclusion criteria were as follows: age 18–75 years; American Society of Anesthesiologists (ASA) I–III, and plan to undergo transabdominal radical gastrectomy for clinical stage I-III gastric cancer. Patients were excluded if their medical records were incomplete or inaccurate, if they had missing abdominal computed tomography (CT), or if they had a history of radical gastric resection or preoperative chemotherapy. Proximal (PG), distal (DG), or total gastrectomy (TG) was performed by specialized surgeons, according to the Japanese Gastric Cancer Treatment Guidelines (23).

### Data collection

For each patient, the data were collected by trained surgeons, radiologists, and anesthesiologists. The surgeons were trained by experienced surgeons until they were sufficiently skilled and precise in data collection (as judged by an experienced surgeon).

The basic information was as follows: patient sociodemographic characteristics, clinical characteristics, surgical procedures, and outcomes. The intraoperative parameters examined were as follows: the types of resection, anesthesia method, operation time (min), mechanical ventilation time (min), respiratory parameters (tidal volume [ml/kg], positive end expiratory pressure [PEEP], airway pressure, end-tidal carbon dioxide), circulation (systolic and diastolic blood pressure, heart rate, intraoperative vasoactive drug use), intraoperative pulse oximetry (SPO_2_) < 95% duration, intraoperative infusion volume (colloid volume, crystalloid volume), urine volume, and medication data.

After surgery, we monitored: the time of removing the tracheal tube after surgery (min), days of hospitalization after surgery (days), hospitalization expenses (yuan), time of removing gastric tube after surgery (days), postoperative complications, and pulmonary complications within 15 days after surgery (24), postoperative destination, whether patient-controlled analgesia was used, postoperative pathological diagnosis, histological type, TNM stage, and readmission within 30 days of discharge.

For primary outcomes, we measured PPCs and for secondary outcome measures, we considered the severity classification of PPCs, other postoperative complications, and intra-abdominal infections.

### Diagnosis of sarcopenia

Sarcopenia was defined as low muscle mass, strength, and/or physical performance. Previous studies have shown that lumbar triplane skeletal muscle index (L3 SMI) on CT is the gold standard for estimating muscle quality (25). After professional training, imaging physicians identified and measured the muscle area of the L3 plane and divided it by the height squared (m^2^) to obtain the skeletal muscle index of L3 SMI (cm^2^/m^2^) on the syngo Multimodality Workplace software (Siemens Medical Solutions, Forchheim, Germany).

**Figure 1:**
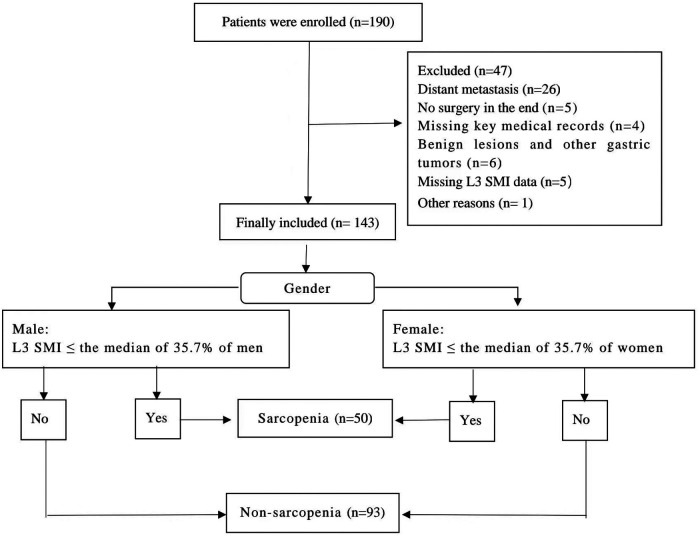
.

Studies have shown that the median prevalence of sarcopenia in patients with gastric cancer is approximately 35.7% (14). Therefore, in this study, we used a median of 35.7% for grouping. In all the medical records collected,  ≤ 35.7% of both sexes were classified as the sarcopenia group and >35.7% as the non-sarcopenia group. A data collection flowchart is presented in Figure 1.

### Complications definition

PPCs were defined as any of the following postoperative conditions within 15 days after operation: initial ventilation support for >48 h, re-intubation due to respiratory failure or pneumonia, respiratory infection, respiratory failure, bronchospasm, atelectasis, pleural effusion, pneumothorax, or aspiration pneumonia (26). The severity of PPCs was classified as 0–5, in which 0 indicates that PPCs have no symptoms or signals, 1–4 indicates gradual deterioration of complications, and 5 indicates death before discharge (27). A grade of at least 2 was defined as severe PPCs. Postoperative complications were defined as any deviation from the normal postoperative course and were graded according to the Clavien-Dindo classification (28).

### Statistical analysis

The Shapiro–Wilk test was used to test the normality of continuous variables. The Student's *t*-test was used for quantitative data of normal and approximately normal distribution, and the mean±standard deviation was used for quantitative data of severely skewed distributions. For such distributions, the rank sum test was used and the data were expressed as median (25% quantile, 75% quantile). Categorical variables were analyzed using the chi-squared or Fisher's exact test, and classified variable data were expressed as numbers and percentages. Univariate logistic regression analysis was used for the univariate analysis. Variables with significant trends and known prognostic values, such as age, were selected as potential parameters in the univariate analysis. Forward stepwise variable selection was used to establish a multivariate logistic regression. All tests were bilateral (except for logistic regression analysis) and were considered statistically significant at *P* < 0.05. The IBM SPSS statistical software version 23.0 (SPSS Inc., Chicago, IL) was used for the statistical analysis.

## Results

### Comparison of baseline characteristics

A total of 143 patients met the inclusion criteria and were included in the study. The basic patient information is summarized in Tables 1, 2. They were divided into sarcopenia and non-sarcopenia groups according to the abdominal CT-guided L3 SMI, and the median prevalence rate was 35.7%.

**Table 1:** Baseline characteristics of sarcopenia vs. non-sarcopenia groups.

	Sarcopenia group (*n* = 50)	Non-sarcopenia group (*n* = 93)	*p*-value
Age (years) (Mean±SD)	59.4 ± 10.1	55.9 ± 10.7	0.064
Gender: Male (%)	33 (66.0)	62 (66.7)	0.936
Height (cm) M (P25, P75)	163.5 (157.8, 170.0)	165.0 (158.0, 170.0)	0.726
Weight (kg) M (P25, P75)	53.3 (47.5, 60.3)	63.0 (58.0, 70.0)	<0.001*
BMI (kg/m^2^) M (P25, P75)	20.0 (18.0, 21.7)	23.2 (21.6,25.8)	<0.001*
Previous history of abdominal surgery (%)	14/36 (28.0/72.0)	19/74 (20.4/79.6)	0.306
Preoperative complication (%)	20 (40.0)	29 (31.2)	0.289
Hypertension (%)	8 (16.0)	12 (12.9)	0.611
Diabetes (%)	4 (8.0)	8 (8.6)	1.000
Chronic lung disease (%)	7 (14.0)	4 (4.3)	0.081
ASA grade (%)			
I	0 (0)	1 (1.1)	
II	36 (72.0)	81 (87.1)	
III	14 (28.0)	10 (10.8)	
SPO_2 _< 95% when inhaling air (%)	1 (2.0)	6 (6.45)	0.434
Surgical approach (Billroth I/RYGB) (%)	21/29 (42.0/58.0)	28/65 (30.1/69.9)	0.153
Surgical types			0.003*
Total gastrectomy	21 (42.0)	29 (31.2)	
Proximal gastrectomy	0 (0)	12 (12.9)	
Distal gastrectomy	29 (58.0)	52 (55.9)	
Pathological diagnosis adenocarcinoma (%)	40 (80.0)	89 (95.7)	0.007*
Histologic Grade (%)			0.783
G2-G3	15 (30.0)	30 (32.2)	
G2	9 (18.0)	15 (16.1)	
G3	16 (32.0)	38 (40.9)	
others	10 (20.0)	10 (10.8)	
TNM Stage			0.143
1	11 (22.0)	31 (35.5)	
2	17 (34.0)	20 (20.4)	
3	22 (44.0)	40 (43.0)	
Neutrophils (10^9^/L) M (P25, P75)	3.0 (2.4, 4.0)	3.1 (2.5, 3.9)	0.912
Lymphocytes (10^9^/L) M (P25, P75)	1.6 (1.3, 1.9)	1.6 (1.3, 1.9)	0.741
Mononuclear cell (10^9^/L) M (P25, P75)	0.4 (0.3, 0.5)	0.4 (0.3, 0.5)	0.599
T-BIL (μmol/L) M (P25, P75)	8.1 (6.4, 10.8)	10.7 (8.3, 14.5)	0.001*
ALT (IU/l) M (P25, P75)	12.5 (9.0, 17.3)	15.0 (12.0, 23.0)	0.006*
AST (IU/L) M (P25, P75)	19.0 (15.0, 22.3)	19.0 (15.5, 23.0)	0.529
Albumin (g/L) Mean ± SD	39.9 ± 4.0	42.3 ± 3.9	0.001*
Blood glucose (mmol/L) M (P25, P75)	4.9 (4.6, 5.3)	5.0 (4.6, 5.4)	0.906
Blood urea nitrogen (mmol/L) M (P25, P75)	4.7 (4.0, 6.0)	4.80 (4.4, 5.5)	0.588
Serum creatinine (μmol/L) Mean ± SD	67.8 ± 15.3	67.9 ± 13.8	0.963
eGFR (ml/min/1.73 m^2^) M (P25, P75)	98.6 (88.7, 104.3)	96.7 (90.5, 104.3)	0.943
Triglyceride (mmol/L) M (P25, P75)	1.1 (0.9, 1.5)	1.3 (0.8, 1.7)	0.175
Total cholesterol (mmol/L) Mean ± SD	4.3 ± 0.8	4.4 ± 0.9	0.396
HDL (mmol/L) M (P25, P75)	1.2 (1.1, 1.5)	1.2 (0.9, 1.4)	0.369
LDL (mmol/L) Mean ± SD	2.5 ± 0.7	2.6 ± 0.7	0.348
LDH (IU/L) Mean ± SD	146.1 ± 28.3	155.6 ± 25.8	0.056
Transferrin (g/L) M (P25, P75)	2.2 (2.0, 2.5)	2.2 (2.0, 2.7)	0.563
Prealbumin (mg/L) M (P25, P75)	204.0 (174.0, 229.0)	222.5 (184.3, 257.5)	0.029*
AFP (ng/ml) M (P25, P75)	2.5 (1.8, 4.0)	3.0 (2.2, 4.1)	0.183
CEA (ng/ml) M (P25, P75)	2.0 (1.3, 3.7)	2.0 (1.1, 3.2)	0.584
CA19-9 (U/ml) M (P25, P75)	10.0 (5.8, 27.4)	10.9 (7.2, 15.2)	0.835
CA-125 (U/ml) M (P25, P75)	14.1 (8.4, 20.1)	12.2 (9.4, 17.1)	0.723

SD, standard deviation; M, median; P25, 25% quantile; P75, 75% quantile; BMI, body mass index; ASA, american society of anesthesiologists classification; SPO_2_, pulse oximetry; RYGB, roux-en-Y gastric bypass; TNM, stage tumor-lymph node-metastasis staging; T-BIL, total bilirubin; ALT, alanine aminotransferase; AST, aspartate aminotransferase; eGFR, estimated glomerular filtration rate; HDL, high density lipoprotein; LDL, low-density lipoprotein; LDH, lactate dehydrogenase; AFP, alpha-fetoprotein; CEA, carcinoembryonic antigen; CA19-9, carbohydrate antigen 19-9; CA-125, carbohydrate antigen 125.

* statistically significant (*P* < 0.05).

**Table 2:** Perioperative management of sarcopenia vs. non-sarcopenia groups.

	Sarcopenia group (*n* = 50)	non-sarcopenia group (*n* = 93)	*p*-value
Operation time (mins) M (P25, P75)	154.0 (130.0, 177.5)	150.0 (130.0, 179.3)	0.957
Colloidal fluid (ml/h) Mean±SD	203.0 ± 129.5	215.0 ± 97.9	0.536
Crystal liquid (ml/h) Mean±SD	555.6 ± 166.2	540.1 ± 165.6	0.595
Urine volume (ml/h/kg) M (P25, P75)	1.5 (0.5, 2.6)	1.3 (0.8,2.1)	0.971
Sevoflurane (ml/h) M (P25, P75)	15.44 (0, 20.9)	15.4 (0, 19.6)	0.662
Desflurane (ml/h) M (P25, P75)	0 (0, 3.9)	0 (0, 6.6)	0.742
Propofol (mg/h) M (P25, P75)	155.9 (32.2, 256.1)	63.6 (32.2, 324.4)	0.563
Dexmedetomidine (ug/h) M (P25, P75)	5.9 (0, 18.2)	14.2 (0, 22.0)	0.065
Sufentanil (ug/h) M (P25, P75)	13.2 (11.4, 15.6)	14.9 (11.8, 16.5)	0.211
Remifentanil (ug/h) Mean±SD	377.0 ± 156.7	446.6 ± 208.0	0.041*
Cisatracurium (mg/h) Mean±SD	8.2 ± 1.8	8.7 ± 2.5	0.181
Use of higher doses of vasoactive drugs (%)	12 (24.0)	17 (18.3)	0.417
Use of high-dose antihypertensive drugs (%)	3 (6.0)	4 (4.3)	0.966
Use higher doses of vasopressors (%)	9 (18.0)	14 (15.1)	0.647
Intraoperative heat preservation (%)	12 (24.0)	18 (19.35)	0.536
Intraoperative blood transfusion (%)	5 (10.0)	4 (4.3)	0.329
Duration of intraoperative SPO_2 _< 95% (mins) M (P25, P75)	0 (0, 0)	0 (0, 0)	0.457
Ventilation strategy Capacitance Control/Voltage Control (%)	28/19 (56.0/38.0)	58/26 (62.4/28.0)	0.465
Mechanical ventilation time (mins) Mean±SD	201.1 ± 35.7	202.8 ± 40.2	0.808
Mean of peep (cmH_2_O) M (P25, P75)	2.9 (2, 3)	2.8 (2, 3)	0.950
Peak airway pressure > 20 cmH_2_O *n* (%)	0 (0)	4 (4.3)	0.335
Peak airway pressure > 15 cm H_2_O duration (mins) M (P25, P75)	0 (0, 15)	0 (0, 45)	0.185
Peak airway pressure > 15 cmH_2_O n (%)	19 (38.0)	42 (45.2)	0.343
Peak airway pressure > 15 cmH_2_O continues to exceed 30 min *n* (%)	9 (18.0)	24 (25.8)	0.262
Peak airway pressure > 15 cmH_2_O continues to exceed 15 min *n* (%)	10 (20.0)	30 (32.3)	0.098
Tidal volume (ml/kg) M (P25, P75)	7.1 (6.3, 8.0)	6.6 (5.8, 7.0)	<0.001*
ETCO_2 _> 45 mmHg duration (mins) M (P25, P75)	0 (0, 5)	0 (0, 0)	0.221
Heart rate change ≥30% duration (mins) M (P25, P75)	7.5 (0, 35.0)	7.5 (0, 25.0)	0.771
Heart rate <55 beats/min duration (mins) M (P25, P75)	10.0 (0,22.5)	5.0 (0, 23.8)	0.979
Heart rate >100 beats/min duration (mins) M (P25, P75)	0 (0,5.0)	0 (0, 5.0)	0.673
SBP change ≥30% duration (mins) M (P25, P75)	12.5 (0, 60.0)	10.0 (1.3, 40.0)	0.787
SBP change ≥20% duration (mins) M (P25, P75)	60.0 (27.5, 112.5)	62.5 (31.3, 105.0)	0.951
DBP change ≥20% duration (mins) M (P25, P75)	65.0 (30.0, 105.0)	60.0 (26.3, 100.0)	0.956
DBP change ≥30% duration (mins) M (P25, P75)	12.5 (0, 36.3)	10.0 (5, 40.0)	0.549
Postoperative destination of the patient (ICU/ inpatient ward) (%)	1/49 (2.0/98.0)	1/92 (1.1/98.9)	
Postoperative analgesia pump (%)	34 (68.0)	76 (81.7)	0.037*

SD, standard deviation; M, median; P25, 25% quantile; P75, 75% quantile; PEEP, positive end expiratory pressure; ETCO_2_, end-tidal carbon dioxide; SBP, systolic blood pressure; DBP, diastolic blood pressure; ICU intensive care unit.

* statistically significant (*P* < 0.05).

Compared with those in the non-sarcopenia group, the patients in the sarcopenia group had lower body weight (kg) (*P* < 0.001), BMI (kg/m^2^) (*P* < 0.001), and preoperative blood albumin (*P* = 0.001). In addition, serum prealbumin (mg/L) (*P* = 0.029), T-BIL (μM) (*P* = 0.001), and ALT (IU/L) (*P* = 0.006) levels were also lower. The intraoperative small dose of remifentanil (µg/h) was lower (*P* = 0.041). The intraoperative tidal volume (ml/kg) of kilogram body weight (ml/kg) in the sarcopenia group was larger than that in the non-sarcopenia group (*P* < 0.001). As for the types of surgical resection, most distal gastrectomies were performed in the two groups, accounting for >50% in each group, while there was no proximal gastrectomy in the sarcopenia group (*P* = 0.003). The postoperative use of an intravenous analgesia pump was lower in the sarcopenia group than in the non-sarcopenia group (*P* = 0.037). In terms of pathological diagnosis, adenocarcinoma was lower in the sarcopenia group than in the non-sarcopenia group (*P* = 0.007). Other preoperative and intraoperative factors were not significantly different between the two groups.

### Comparison of short-term outcomes

Compared with the non-sarcopenia group, the sarcopenia group had worse outcomes for the incidence of the PPCs (*P* = 0.024). Moreover, the incidence of postoperative complications was higher in the sarcopenia group than in the non-sarcopenia group (*P* = 0.043). A total of three patients were then re-admitted within 30 days after discharge (one case in the sarcopenia group and two cases in the non-sarcopenia group). There were no significant differences in hospitalization cost, LOS, postoperative gastric tube extubation time, or postoperative endotracheal tube extubation time (Table 3).

**Table 3:** Outcomes of sarcopenia vs. non-sarcopenia groups.

	Sarcopenia group (*n* = 50)	Non-sarcopenia group (*n* = 93)	*p*-value
Primary outcomes			
PPCs (%)			0.024*
Grade 0	39 (78.0)	85 (91.4)	
Grade 1	0 (0)	2 (2.2)	
Grade 2	6 (12.0)	3 (3.2)	
Grade 3	2 (4.0)	2 (2.2)	
Grade 4	3 (6.0)	1 (1.0)	
Grade 5	0 (0)	0 (0)	
Severe PPCs (≥ Grade 2)	11 (22.0)	6 (6.5)	0.009*
Secondary outcomes			
Postoperative complications (%)			0.043*
Grade 0	32 (64.0)	74 (79.6)	
Grade I	7 (14.0)	11 (11.8)	
Grade II	5 (10.0)	5 (5.4)	
Grade III	3 (6.0)	2 (2.2)	
Grade IV	3 (6.0)	1 (1.0)	
Grade V	0 (0)	0 (0)	
Abdominal infection *n* (%)	4 (8.0)	0 (0)	
Postoperative tracheal tube removal time (mins) M (P25, P75)	12.5 (5.8, 25.3)	10.0 (5.0, 20.0)	0.150
Postoperative gastric tube removal time (days) M (P25, P75)	3.0 (0, 5.0)	3.0 (0, 5.0)	0.451
Length of stay (days) M (P25, P75)	7.0 (6.0, 8.0)	7.0 (6.0, 8.0)	0.684
Number of readmissions within 30 days after discharge *n* (%)	1 (2.0)	2 (2.2)	
Hospital expenses (yuan) M (P25, P75)	81,548.5 (77,895.8, 87,377.8)	79,796.0 (72,411.0, 84,615.0)	0.069

M, median; P25, 25% quantile; P75, 75% quantile; PPCs, postoperative pulmonary complications.

* statistically significant (*P* < 0.05).

### Risk factors of PPCs and postoperative complications

In the univariate analysis, age, sarcopenia, preoperative comorbidities, SPO_2_ < 95% when inhaling air under air, and duration of intraoperative SPO_2_ < 95% were risk factors for the PPCs. In the multivariate analysis that included these factors, sarcopenia (odds ratio [OR] 3.79, 95% confidence interval [CI] 1.27–11.34, *P* = 0.017), preoperative comorbidities (OR 2.86, 95% CI 1.01–8.15, *P* = 0.049), and duration of intraoperative SPO_2_ < 95% (OR 1.14, 95% CI 1.04–1.24. *P* = 0.005) may be risk factors for PPCs. Univariate analysis also revealed that age, sarcopenia, preoperative comorbidities, and the duration of intraoperative SPO_2_ < 95% were risk factors for severe PPCs. Multivariate regression analysis showed that sarcopenia (OR 5.10, 95% CI 1.63–16.00, *P* = 0.005) and the duration of intraoperative SPO_2_ < 95% (OR 1.12; 95% CI 1.02–1.22, *P* = 0.016) were independent risk factors for severe PPCs in patients after gastric cancer surgery (Table 4).

**Table 4:** Univariate and multivariate analysis of factors associated with PPCs.

	PPCs				Severe PPCs			
	Univariate analysis		Multi-factor analysis		Univariate analysis		Multi-factor analysis	
	OR (95% CI)	*p*-value	OR (95% CI)	*p*-value	OR (95% CI)	*p*-value	OR (95% CI)	*p*-value
Age (years)	1.08 1.02–1.15)	0.015*			1.06 (1.00–1.13)	0.047*		
Sarcopenia vs. non-sarcopenia	3.00 (1.12–8.04)	0.029*	3.79 (1.27–11.34)	0.017*	4.09 (1.41–11.85)	0.009*	5.10 (1.63–16.00)	0.005*
Preoperative comorbidities	3.11 (1.16–8.35)	0.024*	2.86 (1.01–8.15)	0.049*	3.19 (1.13–8.99)	0.028*		
SPO_2_ < 95% when inhaling air	5.58 (1.14–27.23)	0.034*			3.20 (0.57–17.97)	0.186		
Duration of intraoperative								
SPO_2_ < 95%	1.11 (1.03–1.21)	0.010*	1.14 (1.04–1.24)	0.005*	1.09 (1.00–1.18)	0.049*	1.12 (1.02–1.22)	0.016*

PPCs postoperative pulmonary complications; SPO_2_ pulse oximetry. Values in parentheses are percentages unless otherwise stated.

* statistically significant (*P* < 0.05).

Univariate analyses also found that sarcopenia was the risk factor for postoperative complications. Multivariate regression analysis with age using binary logistic regression showed that sarcopenia (OR 2.19, 95% CI 1.02–4.72, *P* = 0.045) was an independent risk factor for postoperative complications after gastric cancer surgery (Table 5).

**Table 5:** Univariate and multivariate analysis of factors associated with postoperative complications.

	Univariate analysis		Multi-factor analysis	
	OR (95% CI)	*p*-value	OR (95% CI)	*p*-value
Age (years)	1.03 (0.99–1.08)	0.098		
Sarcopenia vs. non-sarcopenia	2.19 (1.02–4.72)	0.045*	2.19 (1.02–4.72)	0.045*

Values in parentheses are percentages unless otherwise stated. *statistically significant (*P* < 0.05).

## Discussion

We performed a single-center cohort study to investigate the effect of sarcopenia on PPCs in patients after gastric cancer surgery. We found that sarcopenia was associated with a higher incidence of postoperative complications and PPCs, compared to non-sarcopenia, in patients undergoing gastric cancer surgery. Multivariate regression analysis showed that sarcopenia, preoperative comorbidities, and the duration of intraoperative SPO_2_ <95% were the risk factors for PPCs in patients undergoing radical gastrectomy. Sarcopenia and intraoperative SPO_2 _<95% were still the risk factors for severe PPCs. In addition, sarcopenia was an independent risk factor for postoperative complications after gastrectomy.

Some studies have investigated the relationship between sarcopenia and postoperative complications following gastric cancer surgery (29–31). Patients with sarcopenia have a higher risk of postoperative complications and longer LOS. Zhou et al. indicated that sarcopenia is a strong independent risk factor for postoperative complications in older patients with gastric cancer (32). This is consistent with the results of our studies. The mechanism of sarcopenia leading to the increased risk of postoperative complications, especially pulmonary complications, remains unclear, and it is speculated that it may be related to the following possible mechanisms. Respiratory and swallowing muscles are affected by sarcopenia, which can lead to damage to the lungs and swallowing function. Impaired respiratory muscle function and swallowing function may lead to postoperative difficulty in expectoration, aspiration, postoperative pneumonia, and atelectasis (33, 34). Second, sarcopenia is associated with increased insulin resistance and increased circulation of proinflammatory cytokines, which may lead to the risk of postoperative acute lung injury (35). It has been reported that sarcopenia is associated with an increased inflammatory response to surgery (36). Increased inflammatory activity may also lead to pulmonary complications. Muscle fibers produce cytokines and other peptides such as interleukin-6, which affect the immune response by inhibiting the production of tumor necrosis factor-α and insulin resistance (37, 38). Sarcopenia may lead to immune senescence, which is characterized by impaired cellular immune function and increased inflammatory activity (39).

These factors may lead to PPCs. Fortunately, preoperative exercise through inspiratory muscle training, nutritional support, and other preoperative interventions may improve muscle function in patients with sarcopenia and effectively reduce the incidence of PPCs (25, 40). Further prospective studies are required to verify this finding. Sarcopenia can be diagnosed using a questionnaire, action ability test, or L3 SMI on CT. In the future, more attention should be paid to the diagnosis of sarcopenia in different regions, races, and populations, and the relationship between sarcopenia and prognosis should be further explored.

Our study also showed that the longer the duration of SPO_2_ < 95%, the higher the incidence of PPCs, especially the severe PPCs. Previous studies also found that a low SPO_2_ was associated with increased mortality and mortality caused by pulmonary diseases (41). The duration of intraoperative SPO_2_ < 95% may be related to the changes in pulmonary ventilation function caused by lung disease, mechanical ventilation lung injury, operation, and other inflammatory stimulations, which are currently recognized as indicators closely related to PPCs. This study indicated that the management of intraoperative mechanical ventilation could be further improved.

Interestingly, we found that tidal volume was larger in the sarcopenia group (*P* < 0.001) than in the non-sarcopenia group. In clinical practice, the tidal volume is often set according to the patient's body weight and pulmonary function. Compared to the non-sarcopenia group, the sarcopenia group had a smaller body weight but a larger tidal volume. Studies have shown that mechanical ventilation itself can induce inflammation and cooperate with surgery-induced responses. This magnifying inflammatory cascade reaction leads to lung injury and systemic multiple-organ failure.

Sarcopenia negatively affects the prognosis of patients who require mechanical ventilation, increasing all-cause mortality in these patients (42). This may be related to nutritional status, chronic inflammatory reaction, changes in hormone levels, and lack of physical activity in sarcopenia. Some studies have shown that low tidal volume can reduce pulmonary and systemic inflammatory responses compared with conventional tidal volume (43, 44). Mechanical ventilation with a high tidal volume may cause injury in healthy lungs (45, 46). Although the tidal volume of the two groups in this study did not exceed 10 ml/kg, it was higher in the sarcopenia group than in the non-sarcopenia group. There was no significant difference in the average value of PEEP between the two groups (the average value was approximately 2.8–2.9). Some studies have shown that the use of lower levels of PEEP may make the small airways open and close repeatedly, resulting in atelectasis and accelerating the development of pulmonary complications (47, 48). Multifaceted lung-protective ventilation strategies for high-risk patients, combined with low tidal volume, reopening of collapsed alveoli, and moderate levels of PEEP, can prevent further collapse (49). This would help reduce the incidence of postoperative atelectasis, improve clinical results, and reduce the consumption of medical resources. Whether the existing lung-protective ventilation strategy is the best perioperative ventilation management mode for patients with sarcopenia, and how to individualize PEEP and tidal volume to reduce the increase in PPCs caused by mechanical ventilation remain open questions.

Given the adverse effects of sarcopenia on mortality and hospital outcomes, sarcopenia is often considered a treatable indicator in adult respiratory medical treatment (50). It also shows that the prognosis of patients with sarcopenia can be improved through clinical intervention. Previous studies have shown that rehabilitation exercises, nutritional support, and growth hormone supplementation can improve the muscle mass and prognosis of patients with mechanical ventilation (51–53). However, to date, accurate intervention for sarcopenia has been the focus of attention in patients with oligomyopathy. These findings suggest that doctors should pay more attention to the perioperative respiratory system, intraoperative ventilation management, and postoperative lung rehabilitation. It is not limited to the preoperative evaluation and intraoperative management of anesthesiologists, but also includes early identification and intervention by surgeons, rehabilitation doctors, and nurses to reduce its effect on the poor prognosis of patients with sarcopenia. Therefore, it is necessary to carry out a unified standard diagnostic method, larger sample sizes, and multicenter prospective studies on sarcopenia intervention.

Our study bears several limitations. First, this was a single-center retrospective study with incomplete or, in limited cases, absent medical records, and a small sample size. The conclusions of this study need to be verified in additional multicenter prospective studies, involving larger samples. Second, the definitions of sarcopenia were different. In this study, CT-guided L3 SMI was directly used as an index to evaluate sarcopenia, but it was not diagnosed using a muscle strength test. Since this was a retrospective study, we could not comprehensively evaluate skeletal muscle function. Additionally, the cutoff value was not used in this study because of disease type and other factors. The cutoff value depends on measurement techniques, reference studies, and population availability. Moreover, the definition of sarcopenia is greatly influenced by race, population, sex, and other factors. Currently, considerable controversy remains. Therefore, we used the median prevalence rate of patients with gastric cancer to divide the patients into sarcopenia and non-sarcopenia groups. Because some patients visited the local hospital for revisit after surgery, the relevant data for a long time after surgery could not be accurately collected; therefore the long-term prognosis of the patients was not analyzed in this study.

## Conclusions

Our study demonstrates that the duration of intraoperative SPO_2_ <95%, sarcopenia, and preoperative comorbidities were the risk factors for PPCs, especially severe PPCs. Furthermore, sarcopenia was an independent risk factor for postoperative complications. Future large randomized controlled trials and long-term follow-ups are needed to confirm the relationship between sarcopenia and prognosis.

## Data Availability

The original contributions presented in the study are included in the article/Supplementary Material, further inquiries can be directed to the corresponding author/s.

## References

[1] Narici MV Maffulli N. Sarcopenia: characteristics, mechanisms and functional significance. Br Med Bull. (2010) 95:139–59. 10.1093/bmb/ldq008 20200012

[2] Cruz-Jentoft AJ Bahat G Bauer J Boirie Y Bruyère O Cederholm T Sarcopenia: revised European consensus on definition and diagnosis. Age Ageing. (2019) 48(4):601. 10.1093/ageing/afz046 31081853 PMC6593317

[3] Lauretani F Russo CR Bandinelli S Bartali B Cavazzini C Di Iorio A Age-Associated changes in skeletal muscles and their effect on mobility: an operational diagnosis of sarcopenia. J Appl Physiol. (2003) 95(5):1851–60. 10.1152/japplphysiol.00246.2003 14555665

[4] Cawthon PM Marshall LM Michael Y Dam TT Ensrud KE Barrett-Connor E Frailty in older men: prevalence, progression, and relationship with mortality. J Am Geriatr Soc. (2007) 55(8):1216–23. 10.1111/j.1532-5415.2007.01259.x 17661960

[5] Douglas PJ Short KR Campbell WW Elena V Wolfe RR. Role of dietary protein in the sarcopenia of aging. Am J Clin Nutr (2008) (5):1562S. 10.1093/ajcn/87.5.1562S 18469288

[6] Sayer AA Dennison EM Syddall HE, Jameson K, Martin HJ, Cooper C. The developmental origins of sarcopenia: using peripheral quantitative computed tomography to assess muscle size in older people. J Gerontol-Biol Sci Med Sci. (2008) 63(8):835–40. 10.1093/gerona/63.8.835 PMC2652118 18772471

[7] Dodson S Baracos VE Jatoi A Evans WJ Cella D Dalton JT Muscle wasting in cancer cachexia: clinical implications, diagnosis, and emerging treatment strategies. Annu Rev Med. (2011) 62(1):265. 10.1146/annurev-med-061509-131248 20731602

[8] Stojcev Z Matysiak K Duszewski M Banasiewicz T. The role of dietary nutrition in stomach cancer. Contemp Oncol. (2013) 17(4):343–5. 10.5114/wo.2013.37213 PMC3934052 24592120

[9] Takiguchi S Takata A Murakami K Miyazaki Y Yanagimoto Y Kurokawa Y Clinical application of ghrelin administration for gastric cancer patients undergoing gastrectomy. Gastric Cancer. (2014) 17(2):200. 10.1007/s10120-013-0300-8 24253567

[10] Siegel RL Miller KD Jemal A. Cancer statistics, 2016. CA: Cancer J Clin. (2016) 66(1):7–30. 10.3322/caac.21332 26742998

[11] Tamura T Sakurai K Nambara M Miki Y Toyokawa T Kubo N Adverse effects of preoperative sarcopenia on postoperative complications of patients with gastric cancer. Anticancer Res. (2019) 39(2):987–92. 10.21873/anticanres.13203 30711985

[12] MorleyJE. Sarcopenia: diagnosis and treatment. *J Nutr Health Aging*. (2008) 12(7):452–6. 10.1007/BF0298270518615226

[13] Cheng Q Zhu X Zhang X Li H Du Y Hong W A cross-sectional study of loss of muscle mass corresponding to sarcopenia in healthy Chinese men and women: reference values, prevalence, and association with bone mass. J Bone Miner Metab. (2014) 32(1):78–88. 10.1007/s00774-013-0468-3 23620096

[14] McGovern J Dolan RD Horgan PG Laird BJ McMillan DC. Computed tomography-defined low skeletal muscle Index and density in cancer patients: observations from a systematic review. J Cachexia Sarcopenia Muscle. (2021) 12(6):1408–17. 10.1002/jcsm.12831 34664431 PMC8718024

[15] Kota K Hiroko YS. Comparison of time trends in stomach cancer incidence (1973–2002) in Asia, from cancer incidence in five continents, vols iv–ix. Jpn J Clin Oncol. (2009) 39(1):71–2. 10.1093/jjco/hyn150 19103673

[16] Thrumurthy SG, Chaudry MA, Hochhauser D, Mughal M. The diagnosis and management of gastric cancer. *BMJ*. (2013) 347, f6367. 10.1136/bmj.f6367 24191271

[17] Takeshita H Ichikawa D Komatsu S Kubota T Okamoto K Shiozaki A Surgical outcomes of gastrectomy for elderly patients with gastric cancer. World J Surg. (2013) 37(12):2891–8. 10.1007/s00268-013-2210-7 24081528

[18] Zhou CJ Chen FF Zhuang CL Pang WY Zhang FY Huang DD Feasibility of radical gastrectomy for elderly patients with gastric cancer. Eur J Surg Oncol. (2016) 42(2):303–11. 10.1016/j.ejso.2015.11.013 26710992

[19] Yan-Mei B Xin-Zu C Cheng-Kun J Ru-Bai Z Yu-Fei G Li-Bo Y Safety and survival benefit of surgical management for elderly gastric cancer patients. Hepato-gastroenterol. (2014) 61(134):1801–5. 25436382

[20] Wang SL Zhuang CL Huang DD Pang WY Lou N Chen FF Sarcopenia adversely impacts postoperative clinical outcomes following gastrectomy in patients with gastric cancer: a prospective study. Ann Surg Oncol. (2016) 23(2):556–64. 10.1245/s10434-015-4887-3 26668085

[21] Gertsen EC Goense L Brenkman HJF van Hillegersberg R Ruurda JP. Identification of the clinically most relevant postoperative complications after gastrectomy: a population-based cohort study. Gastric Cancer. (2020) 23(2):339–48. 10.1007/s10120-019-00997-x 31482476 PMC7031165

[22] Wong JU Tai FC Huang CC. An examination of surgical and survival outcomes in the elderly (65-79 years of age) and the very elderly (≥80 years of age) who received surgery for gastric cancer. Curr Med Res Opin. (2020) 36(2):229–33. 10.1080/03007995.2018.1520083 31841040

[23] Suo J Wei LI. Interpretation of Japanese gastric cancer association(jgca) gastric cancer treatment guidelines 2018-the 5th edition. Chin J Pract Surg. (2018) 24(1):1–21. 10.1007/s10120-020-01042-y PMC7790804 32060757

[24] Clavien PA Barkun J Oliveira MLD Vauthey JN Makuuchi M. The clavien-dindo classification of surgical complications: five-year experience. Ann Surg. (2009) 250(2):187. 10.1097/SLA.0b013e3181b13ca2 19638912

[25] Phillips SM. Nutritional supplements in support of resistance exercise to counter age-related sarcopenia. Adv Nutr. (2019) 6(4):452–60. 10.3945/an.115.008367 PMC4496741 26178029

[26] Canet J Gallart L Gomar C Paluzie G Vallès J Castillo J Prediction of postoperative pulmonary complications in a population-based surgical cohort. Anesthesiol. (2010) 113(6):1338. 10.1097/ALN.0b013e3181fc6e0a 21045639

[27] Hulzebos EH Helders PJ Favié NJ De Bie RA Brutel de la Riviere A Van Meeteren NL. Preoperative intensive inspiratory muscle training to prevent postoperative pulmonary complications in high-risk patients undergoing cabg surgery: a randomized clinical trial. Jama. (2006) 296(15):1851–7. 10.1001/jama.296.15.1851 17047215

[28] Dindo D Demartines N Clavien PA. Classification of surgical complications: a new proposal with evaluation in a cohort of 6336 patients and results of a survey. Ann Surg. (2004) 240(2):205–13. 10.1097/01.sla.0000133083.54934.ae 15273542 PMC1360123

[29] Yang Z Zhou X Ma B Xing Y Jiang X Wang Z. Predictive value of preoperative sarcopenia in patients with gastric cancer: a meta-analysis and systematic review. J Gastrointest Surg. (2018) 22(11):1890–902. 10.1007/s11605-018-3856-0 29987739

[30] Kuwada K Kuroda S Kikuchi S Yoshida R Nishizaki M Kagawa S Sarcopenia and comorbidity in gastric cancer surgery as a useful combined factor to predict eventual death from other causes. Ann Surg Oncol. (2018) 25(5):1160–6. 10.1245/s10434-018-6354-4 29404820 PMC5891547

[31] Fukuda Y Yamamoto K Hirao M Nishikawa K Nagatsuma Y Nakayama T Sarcopenia is associated with severe postoperative complications in elderly gastric cancer patients undergoing gastrectomy. Gastric Cancer. (2016) 19(3):986–93. 10.1007/s10120-015-0546-4 26407875

[32] Zhou CJ Zhang FM Zhang FY Yu Z Chen XL Shen X Sarcopenia: a new predictor of postoperative complications for elderly gastric cancer patients who underwent radical gastrectomy. J Surg Res. (2017) 211:137. 10.1016/j.jss.2016.12.014 28501109

[33] Wakabayashi H Sakuma K. Rehabilitation nutrition for sarcopenia with disability: a combination of both rehabilitation and nutrition care management. J Cachexia Sarcopenia Muscle. (2014) 5(4):269–77. 10.1007/s13539-014-0162-x 25223471 PMC4248414

[34] Bahat G Tufan A Ozkaya H Tufan F Akpinar TS Akin S in Male nursing home residents. Aging Male. (2014) 17(3):136–40. 10.3109/13685538.2014.936001 24993454

[35] Nishigori T Okabe H Tanaka E Tsunoda S Sakai Y. Sarcopenia as a predictor of pulmonary complications after esophagectomy for thoracic esophageal cancer. J Surg Oncol. (2016) 113(6):678–84. 10.1002/jso.24214 26936808

[36] Reisinger KW Derikx JPM Vugt JLAV Meyenfeldt MFV Hulsewé KW Damink SWMO Sarcopenia is associated with an increased inflammatory response to surgery in colorectal cancer. Clin Nutr. (2016) 35(4):924–7. 10.1016/j.clnu.2015.07.005 26205321

[37] Pedersen BK Febbraio MA. Muscles, exercise and obesity: skeletal muscle as a secretory organ. Nat Rev Endocrinol. (2012) 8(8):457–65. 10.1038/nrendo.2012.49 22473333

[38] Pedersen BK Bruunsgaard H. Possible beneficial role of exercise in modulating low-grade inflammation in the elderly. Scand J Med Sci Sports. (2003) 13(1):56–62. 10.1034/j.1600-0838.2003.20218.x 12535318

[39] Bruunsgaard H Pedersen BK. Effects of exercise on the immune system in the elderly population. Immunol & Cell Biol. (2000) 78(5):523–31. 10.1046/j.1440-1711.2000.00965.x 11050535

[40] N S. Preoperative inspiratory muscle training and postoperative complications. J Am Med Assoc JAMA. (2007) 297(7):697–9. 10.1001/jama.297.7.697-a 17312285

[41] Vold ML Aasebø U Wilsgaard T Melbye H. Low oxygen saturation and mortality in an adult cohort: the tromsø study. BMC Pulm Med. (2015) 15:9. 10.1186/s12890-015-0003-5 25885261 PMC4342789

[42] Jiang T Lin T Shu X Song Q Dai M Zhao Y Prevalence and prognostic value of preexisting sarcopenia in patients with mechanical ventilation: a systematic review and meta-analysis. Critical Care. (2022) 26(1):140. 10.1186/s13054-022-04015-y 35578299 PMC9109453

[43] Michelet P D'Journo XB Roch A Doddoli C Marin V Papazian L Protective ventilation influences systemic inflammation after esophagectomy: a randomized controlled study. Anesthesiol. (2006) 105(5):911–9. 10.1097/00000542-200611000-00011 17065884

[44] Determann RM Royakkers A Wolthuis EK Vlaar AP Choi G Paulus F Ventilation with lower tidal volumes as compared with conventional tidal volumes for patients without acute lung injury: a preventive randomized controlled trial. Critical Care. (2010) 14(1):R1. 10.1186/cc8230 20055989 PMC2875503

[45] Weingarten TN Whalen FX Warner DO Gajic O Schears GJ Snyder MR Comparison of two ventilatory strategies in elderly patients undergoing Major abdominal surgery. Br J Anaesth. (2010) 104(1):16–22. 10.1093/bja/aep319 19933173

[46] Neto AS Cardoso SO Manetta JA Pereira VGOM Espósito DC Manoela DOPP Association between use of lung-protective ventilation with lower tidal volumes and clinical outcomes among patients without acute respiratory distress syndromes. Surv Anesthesiol. (2014) 58(3):108–9. 10.1097/01.SA.0000446366.05578.70 23093163

[47] Benidixen HH Hedley-Whyte J Laver MB. Impaired oxygenation in surgical patients during general anesthesia with controlled ventilation. A concept of atelectasis. Surv Anesthesiol. (1964) 8(6):571. 10.1056/NEJM196311072691901 14059732

[48] Duggan M Kavanagh BP. Pulmonary atelectasis: a pathogenic perioperative entity. Anesthesiol. (2005) 102(4):838–54. 10.1097/00000542-200504000-00021 15791115

[49] Futier E Constantin JM Pelosi P Chanques G Jaber S. Noninvasive ventilation and alveolar recruitment maneuver improve respiratory function during and after intubation of morbidly obese patients: a randomized controlled study. Anesthesiol. (2011) 114(6):1354–63. 10.1097/ALN.0b013e31821811ba 21478734

[50] McDonald VM Osadnik CR Gibson PG. Treatable traits in acute exacerbations of chronic airway diseases. Chron Respir Dis. (2019) 16:1479973119867954. 10.1177/1479973119867954 31409129 PMC6696844

[51] Medrinal C Combret Y Prieur G Robledo Quesada A Bonnevie T Gravier FE Comparison of exercise intensity during four early rehabilitation techniques in sedated and ventilated patients in icu: a randomised cross-over trial. Critical Care. (2018) 22(1):110. 10.1186/s13054-018-2030-0 29703223 PMC5923017

[52] van Zanten ARH De Waele E Wischmeyer PE. Nutrition therapy and critical illness: practical guidance for the icu, post-icu, and long-term convalescence phases. Critical Care. (2019) 23(1):368. 10.1186/s13054-019-2657-5 31752979 PMC6873712

[53] Hermans G De Jonghe B Bruyninckx F Van den Berghe G. Interventions for preventing critical illness polyneuropathy and critical illness myopathy. Cochrane Database Syst Rev. (2009) 1:Cd006832. 10.1002/14651858.CD006832.pub2 19160304

